# Diffusion tensor imaging of formalin fixed infarcted porcine hearts: a comparison between 3T and 1.5T

**DOI:** 10.1186/1532-429X-15-S1-W34

**Published:** 2013-01-30

**Authors:** R Mazumder, S Choi, B Raterman, BD Clymer, A Kolipaka, RD White

**Affiliations:** 1 Department of Electrical and Computer Engineering, The Ohio State University, Columbus, OH, USA; 2 Department of Radiology, The Ohio State University Wexner Medical Center, Columbus, OH, USA; 3 Department of Internal Medicine, Division of Cardiology, The Ohio State University Wexner Medical Center, Columbus, OH, USA

## Background

Diffusion Tensor Imaging (DTI) quantifies the amount of anisotropic diffusion exhibited by biological tissues. Processing DTI images allow a 3D visualization of the fiber architecture by tracking the fiber trajectories within the tissue. Experimental evidence has shown that the myocardium undergoes remodeling as myocardial infarction progresses over time[1]. The aim of this study is to investigate and compare the fiber architecture in an infarcted porcine heart using DTI at 1.5T and 3T, to analyze the effect of high field magnets in imaging.

## Methods

**Figure 1:**
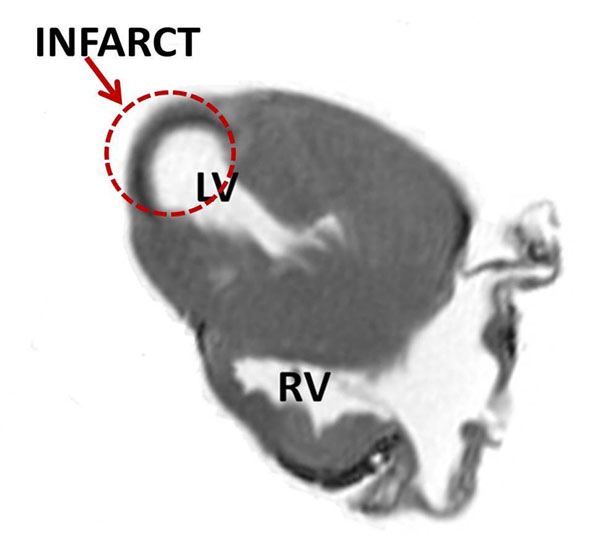
Magnitude image of the infarcted porcine heart, showing the infarcted region near the apex.

Ex-vivo DTI was performed on an infracted pig heart on 1.5T (Avanto, Siemens Healthcare, Germany) and 3T (Tim Trio, Siemens Healthcare, Germany) MRI scanners. Infarcts were created in the apex region (Fig 1) by occluding the left anterior descending coronary artery. After 22 days, the hearts were dissected and formalin fixed for 6 months. A diffusion-weighted echo planar imaging sequence was used to acquire multi-slice short axis views covering the ventricles in the excised heart. Imaging parameters included: diffusion encoding directions=256; TE=90ms; TR=7000(1.5T), 6600(3T) ms; slice thickness=2mm; matrix=128x128; FOV=256x256mm2; b-values=0,1000s/mm2; slices=37(1.5T), 42(3T); isotropic resolution of 2x2x2mm. The images were masked to segment the left ventricular myocardium (LVM). Explore DTI [2], was used to obtain a tensor map and track the fibers using a deterministic algorithm. For this analysis, fractional anisotropy (FA) and the angle between the longest eigenvectors (V1) of the two successive voxels were set to 0.2 and 45 degrees respectively. The lower limit of the length of the fibers was varied from 2mm to 30mm to see the corresponding change in fiber tracts near the infracted region of the LVM obtained from both 1.5T and 3T scanners.

## Results

**Figure 2:**
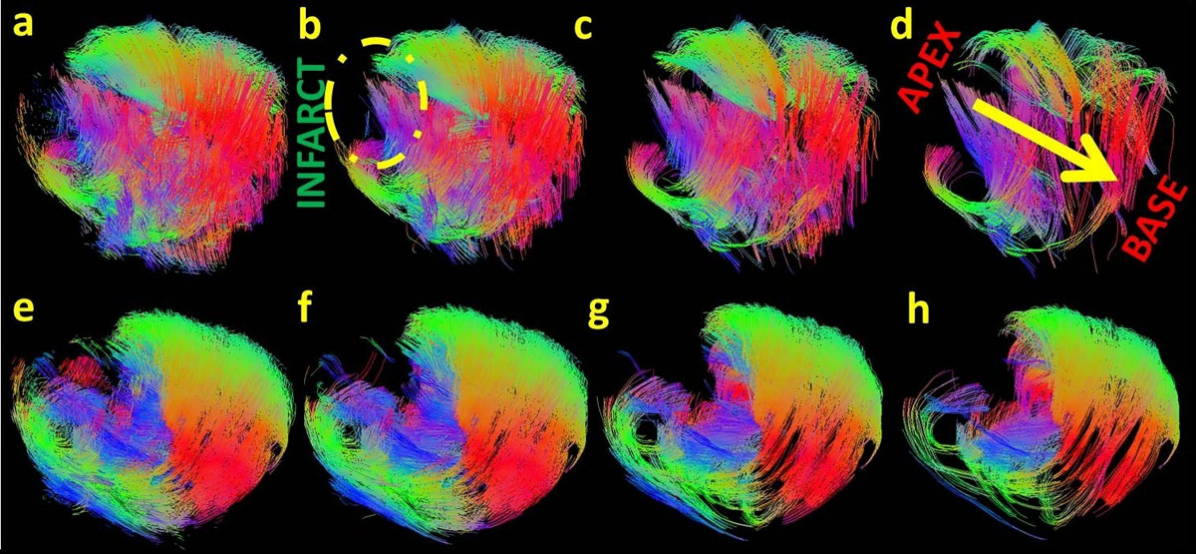
3D volumetric representation of fiber tracts in an infarcted porcine myocardium. First row (a-d) and second row (e-h) displays data from 3T and 1.5T MRI scanners respectively. In a row, left indicates 4 different ranges of fibers tracked. a),e) 2-500 mm b),f) 10-500mm c),g) 20-500 mm and d),h) 30-500 mm. The direction of the yellow arrow on d indicates the progression from apex to base of the myocardium. The color code denotes the direction of fiber orientation; Green, red and blue corresponds to the x, y and z directions of the image respectively. At the apex, we observe disarrayed shorter fibers identifying the infarcted region and the fibers eventually vanish with increase in the lower limit of the fiber length from left to right in both the datasets.

Fig 1 shows the magnitude image displaying the infarct with thin myocardial wall. Fig 2 displays 3D visualization of the fiber tracts in the LVM. In Fig 2 the upper row and the lower row displays data from 3T and 1.5T scanners respectively. From left to right the lower limit of the fiber length was varied in the analysis to track the short disarrayed fibers near the infarct (the apex of the heart). Comparing the extreme left column (tracking length range: 2-500 mm) to the extreme right column (tracking length range: 30-500 mm), we see that more fibers are tracked at the apex of the heart when shorter fiber lengths are included.

## Conclusions

From this preliminary study we observe that the fibers from both the scanners are consistent in terms of the lengths being short and disarrayed around the infracted region compared to the rest of the LVM. We also observe that in the data from the 1.5T scanner fibers tracked are smoother and more in number compared to the data from the 3T scanner. However, more studies are warranted to confirm our analogy and establish the technique.

## Funding
